# The biomechanical fundamentals of crosslink-augmentation in posterior spinal instrumentation

**DOI:** 10.1038/s41598-022-11719-2

**Published:** 2022-05-10

**Authors:** Frédéric Cornaz, Marie-Rosa Fasser, Jess Gerrit Snedeker, José Miguel Spirig, Mazda Farshad, Jonas Widmer

**Affiliations:** 1 Department of Orthopedics, Balgrist University Hospital, University of Zurich, Zurich, Switzerland; 2 Institute for Biomechanics, ETH Zurich, Zurich, Switzerland; 3 Spine Biomechanics, Department of Orthopedics, Balgrist University Hospital, University of Zurich, Zurich, Switzerland

**Keywords:** Medical research, Musculoskeletal system

## Abstract

Posterior screw-rod constructs can be used to stabilize spinal segments; however, the stiffness is not absolute, and some motion can persist. While the effect of crosslink-augmentation has been evaluated in multiple studies, the fundamental explanation of their effectiveness has not been investigated. The aim of this study was to quantify the parameters “screw rotation” and “parallelogram deformation” in posterior instrumentations with and without crosslinks to analyze and explain their fundamental effect. Biomechanical testing of 15 posteriorly instrumented human spinal segments (Th10/11—L4/L5) was conducted in axial rotation, lateral bending, and flexion–extension with ± 7.5 Nm. Screw rotation and parallelogram deformation were compared for both configurations. Parallelogram deformation occurred predominantly during axial rotation (2.6°) and was reduced by 60% (−1.45°, p = 0.02) by the addition of a crosslink. Simultaneously, screw rotation (0.56°) was reduced by 48% (−0.27°, p = 0.02) in this loading condition. During lateral bending, 0.38° of parallelogram deformation and 1.44° of screw rotation was measured and no significant reduction was achieved by crosslink-augmentation (8%, −0.03°, −p = 0.3 and −13%, −0.19°, p = 0.7 respectively). During flexion–extension, parallelogram deformation was 0.4° and screw rotation was 0.39° and crosslink-augmentation had no significant effect on these values (−0.12°, −30%, p = 0.5 and −0°, −0%, p = 0.8 respectively). In axial rotation, crosslink-augmentation can reduce parallelogram deformation and with that, screw rotation. In lateral bending and flexion–extension parallelogram deformation is minimal and crosslink-augmentation has no significant effect. Since the relatively large screw rotation in lateral bending is not caused by parallelogram deformation, crosslink-augmentation is no adequate countermeasure. The fundamental understanding of the biomechanical effect of crosslink-augmentation helps better understand its potential and limitations in increasing construct stiffness.

## Introduction

**Figure 1:**
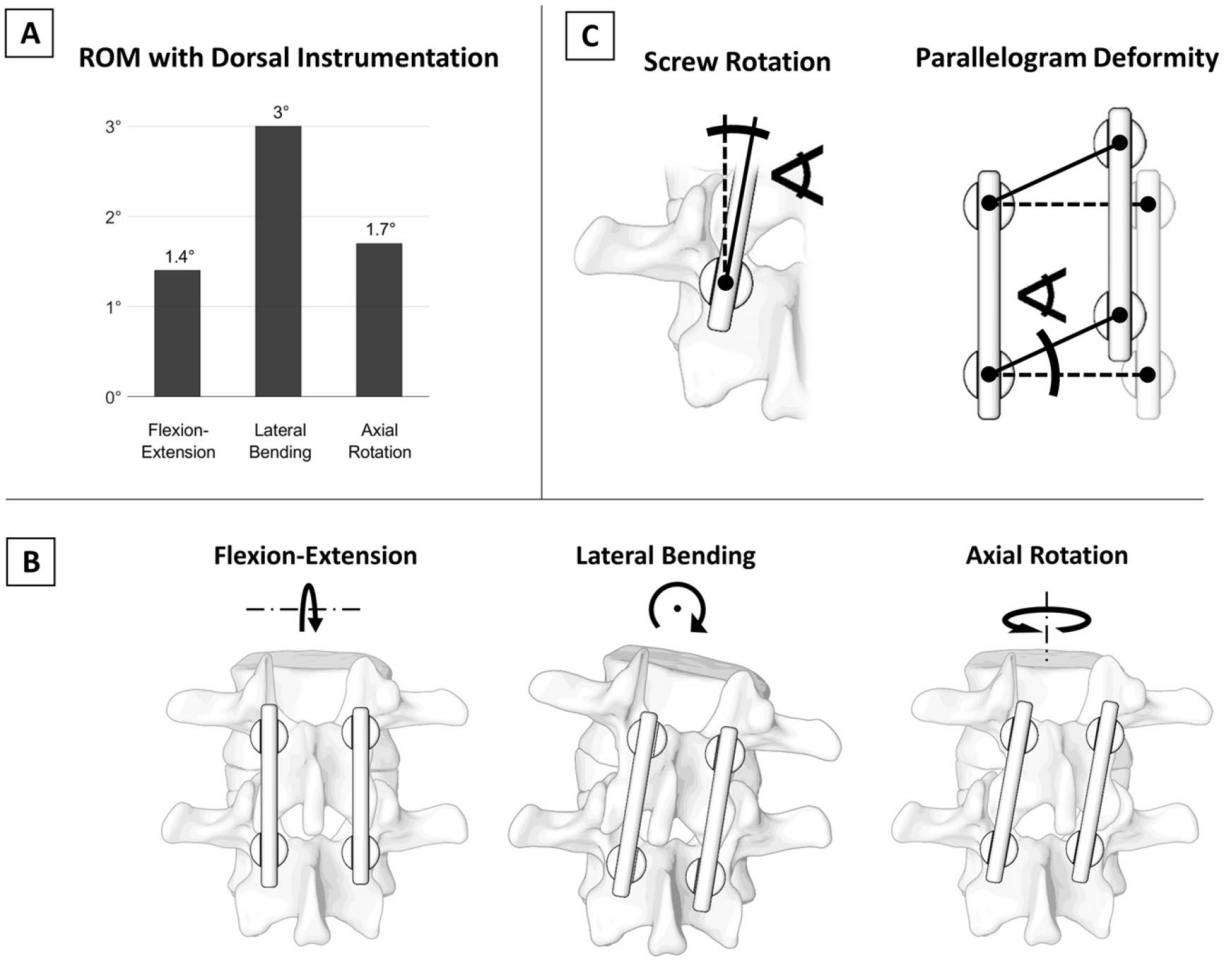
(**A**) Segmental deformation after posterior instrumentation during physiological loading (± 7.5 Nm)^1^. (**B**) Hypothezides construct deformations due to the bending forces in the three major rotational motion planes (figure adapted from^3^). (**C**) Illustration of the angular displacement of the pedicle screw in relation to the vertebral body (“screw rotation”) and the relative motion between one side of the screw-rod-construct to the other (“parallelogram deformation”).

During physiological loading, notable deformation of posteriorly instrumented spinal segments can occur (Fig. 1A)^1^, which can be a relevant risk factor for delayed union or failed bony fusion resulting in unfavorable surgical outcome^2^. The acting bending moments can be separated into three major rotational motion planes: flexion–extension, lateral bending and axial rotation. The hypothezides construct deformation due to the generated internal stresses are illustrated in Fig. 1B. The resulting motion could be caused by a combination of bone and construct deformation, angular displacement at the screw-bone-interface (“screw rotation”, Fig. 1C), and relative displacement of the two sides of the construct (“parallelogram-deformation”, Fig. 1C).

Crosslinks, connecting the two sides of the screw-rod construct, were proposed as a measure to increase rotational construct stiffness^4^ and their effect was evaluated in a multitude of studies, as summarized in a recent systematic review^3^: in axial rotation, a relatively consistent positive effect on construct stiffness was observed, while the effect on lateral bending was more variable and in flexion–extension, only minimal effect was recorded. While the biomechanical effect was largely similair for the whole spinal column, clinical benefit has only been shown for C1/2 instrumentations^5,6^. For the posterior instrumentation in adolescent idiopathic scoliosis, no clinical benefit was shown^7,8^ and no clinical data is available for other clinical situations at the lumbar or thoracic spine. To help direct clinical studies and the clinical practice towards the optimal use of crosslinks, a more fundamental understanding of the biomechanical effect on the construct charachteristics is required.

We hypothesize that while relevant screw rotation and parallelogram-deformation can occur in posterior screw-rod instrumentations, crosslinks are only able to reduce parallelogram-deformation and are ineffective in reducing screw rotation. To quantify the biomechanical effect of crosslink-augmentation on these parameters, posteriorly instrumented single-level human segments were analyzed during axial rotation, lateral bending, and flexion–extension.

## Materials and methods

The experimental protocol was approved by the local ethics institution (Swissethics, BASEC Nr. 2017-00874) and conforms to the relevant guidelines and regulations. All medical information about the donors was fully anonymized and the original written informed consent for donation, in accordance with applicable law and regulation are on file at the offices of Science Care (Science Care, Phoenix, AZ, USA). Biomechanical experiments were performed on 15 human spinal segments (Th10/11—L4/L5) originating from four fresh frozen cadavers (Table 1).

**Table 1:** Overview on the spinal segments used for this study and the pedicle screw rajectories used for instrumentation.

#	Demographics	Th9/10	Th10/11	Th11/12	Th12/L1	L1/2	L2/3	L3/4	L4/5
1	65 years, female	–	TT	–	CBT	–	TT	–	CBT
2	45 years, female	–	CBT	–	TT	–	CBT	–	TT
3	62 years, male	CBT	–	TT	–	CBT	–	TT	–
4	64 years, male	Excluded	–	CBT	–	TT	–	CBT	–

*TT* traditional trajectory, *CBT* cortical bone trajectory.

**Figure 2:**
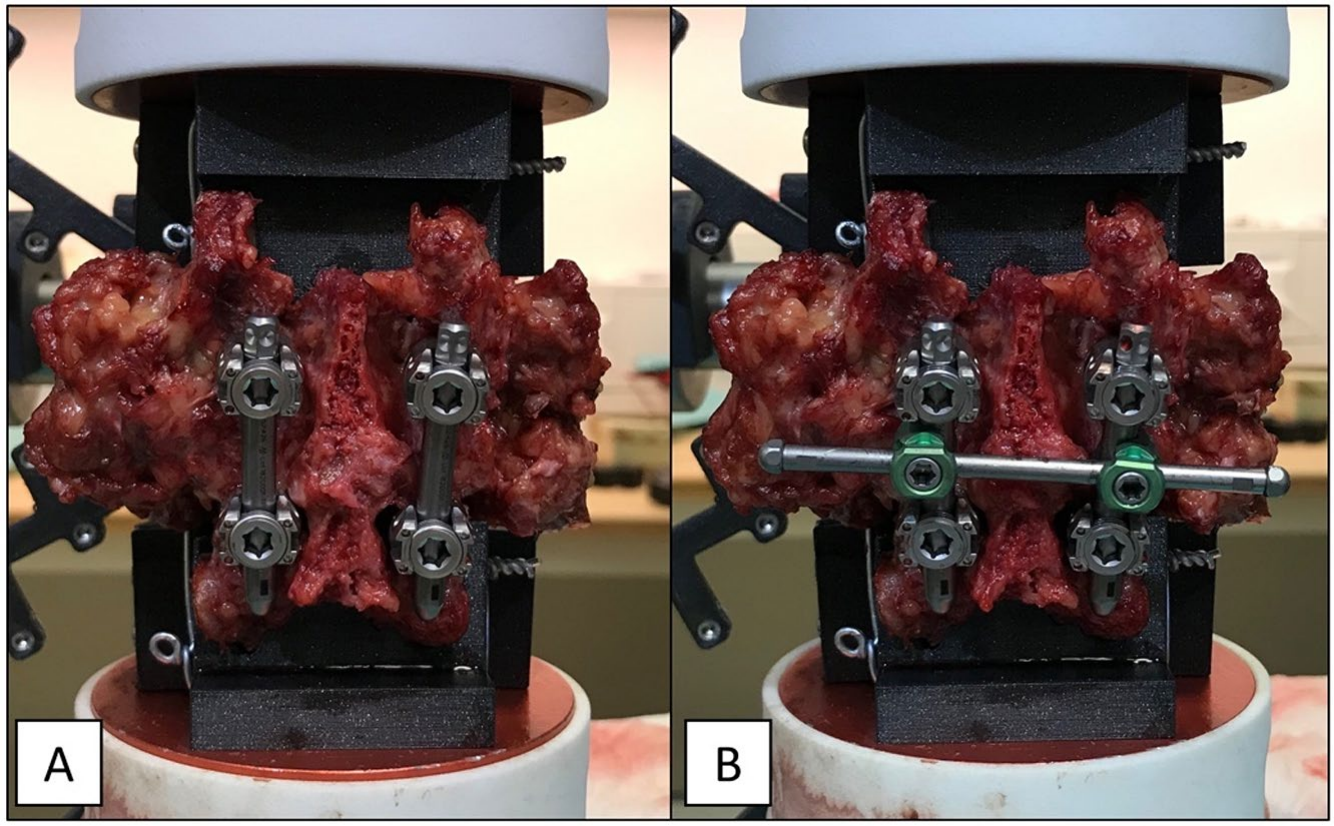
Image of a dorsally instrumented spinal segment (**A**) without and (**B**) with crosslink-augmentation.

Directly before the here reported experiment, the range of motion of the spinal segments with and without posterior instrumentations was measured under quasi-physiological loading conditions and the results as well as further specification of the specimens are reported elsewhere^1^. Testing duration of the previous testing was below 2 h per specimen, acted as preconditioning. The here reported experiments took about 30 min per specimen. The spinal segments were instrumented with cannulated pedicle screws following the traditional (7 segments) or the cortical bone trajectory (8 segments)^9^ with specific insertion guides^10^ . Screw diameter and length were maximized during preoperative planning^11^ and ranged from 5 to 6 mm in diameter and 40 mm to 55 mm in length (M.U.S.T, Medacta International, Switzerland). Vertical rods (pre-bent rods, titanium 5.5 × 50 mm, ref. 03.50.453, M.U.S.T, Medacta International, Switzerland) were inserted on either side (Fig. 2A). A commercially available crosslink was used for the experiments (straight cross connector, ref. 03.56.408, M.U.S.T Medacta International, Switzerland) (Fig. 2B).

**Figure 3:**
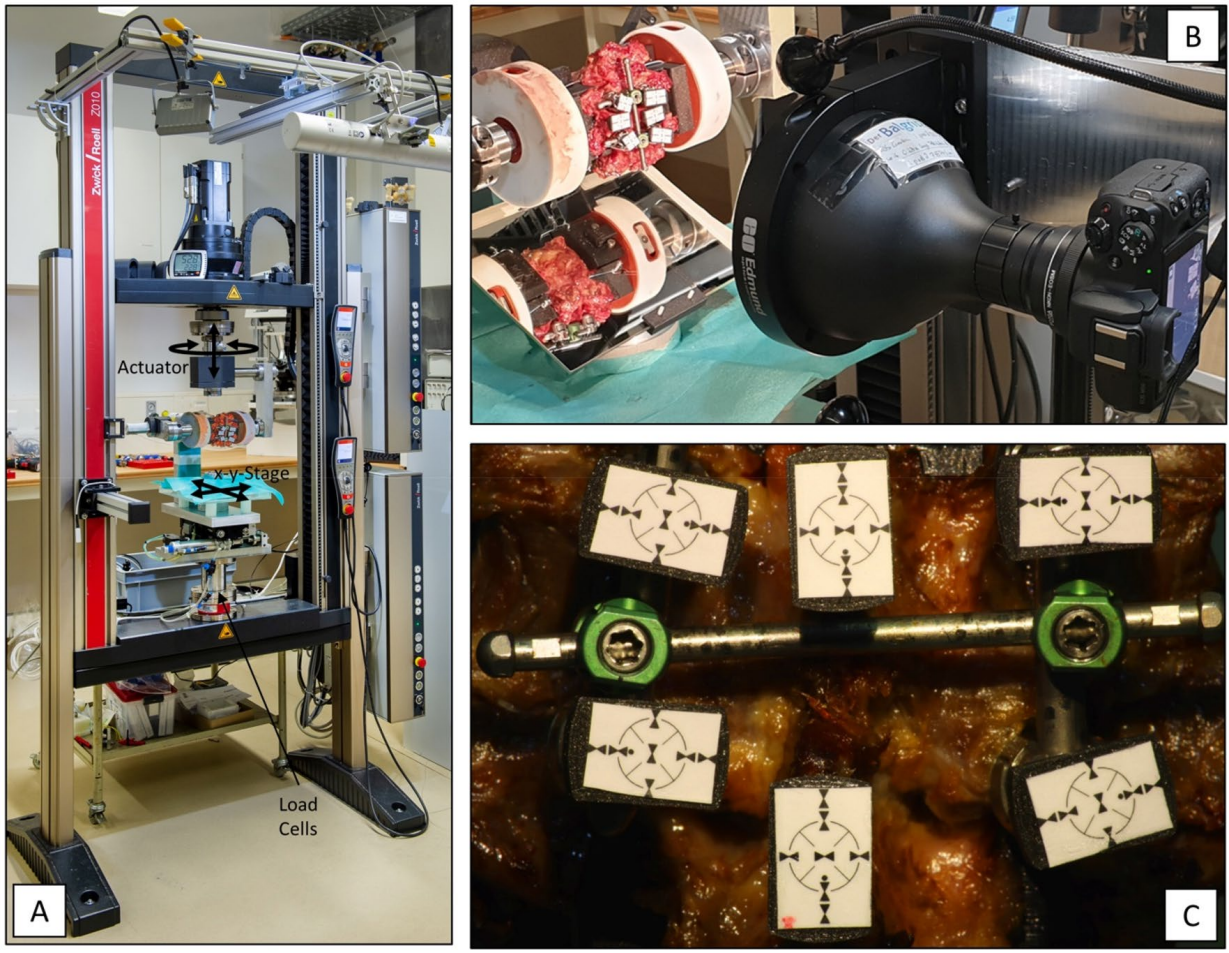
(**A**) Image of the biomechanical test setup with the mounting position to perform flexion–extension loading. (**B**) Telecentric camera system. (**C**) Telecentric image of an illustrative specimen with the six crosshair-labels to measure translational and rotational displacement of the two vertebral bodies and the four screw heads.

A spine testing machine (Zwick/Roell Allroundline 10kN and testXpert III Software, ZwickRoell GmbH & Co. KG, Germany) with a specific spine testing setup^1,12–14^ (Fig. 3A) was used to apply bending moments of ± 7.5 Nm in axial rotation, lateral bending, and flexion–extension. To generate the desired bending motions, the specimens were reoriented in the test machine^12^. Loading speed was 1°/s and the 7.5 Nm loading amplitude was actively held for 10 s, during which imaging was performed. Translational coupled motion in the plane orthogonal to the rotation axis was left unconstrained with the use of an x–y-table^1^. Testing was performed at room temperature and the specimens were frequently sprayed with phosphate buffered saline (PBS) to prevent dehydration. A camera system with a telecentric objective (Edmund Optics #62-921, 182 mm WD, 0.28X, Edmund Optics Inc., Barrington, NJ, USA) was used to image the screw-rod construct in the configuration with and without crosslink-augmentation for each loading direction (Fig. 3B).

To image the deformation during lateral bending (in which the instrumentation faced downwards), a mirror oriented at 45° relative to the floor was used (Fig. 3B). The testing sequence (loading condition and crosslink addition) was reversed for half of the segments.

For every segment, each of the four screw heads and the two vertebral bodies were furnished with a specifically designed crosshair-label that allowed for the assessment of the rotational and translational position in the obtained pictures (Fig. 3C). Image processing was performed automatically with a specifically developed script in MATLAB (Matlab R2019a, Mathworks Inc.). The position and rotation of every crosshair-label were detected, and perspective errors were corrected by considering the ellipsity of the label on the 2D image. Screw rotation was measured by averaging the relative rotation between the cranial screw-heads and the cranial vertebra and between the caudal screw-heads and the caudal vertebra. Parallelogram-deformation was defined as the pooled difference in angle between the left screw heads in relation to the caudal screw heads and the right screw heads in relation to the caudal screw heads.

Statistical evaluation was performed with MATLAB. According to the Shapiro–Wilk parametric hypothesis tests of composite normality, not all results were normally distributed. Therefore, the Wilcoxon signed-rank test was used (α = 0.05).

## Results

**Figure 4:**
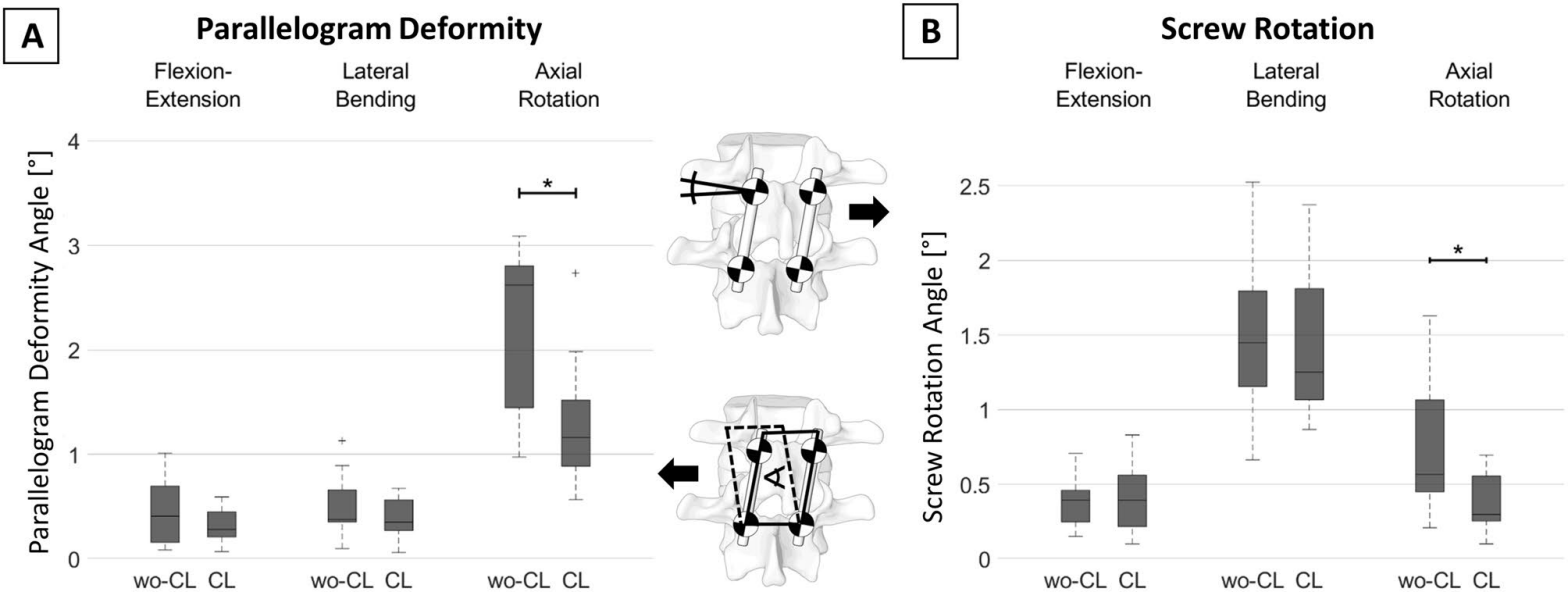
Effect on (**A**) parallelogram deformation and (**B**) screw rotation by the addition of a crosslink to the construct. Significant differences (p < 0.05) are marked with an asterisk (*), *wo-CL* configuration without crosslink, *CL*  configuration with crosslink.

All data generated or analysed during this study are included in this published article (and its supplementary information files). Analysing all 15 spinal segments combined, the median value of the parallelogram deformation without crosslink was 2.6° in axial rotation, 0.38° in lateral bending and 0.4° in flexion–extension. The addition of a crosslink reduced this value to 1.15° in axial rotation (−1.45°, −60%, p = 0.02), to 0.35° in lateral bending (−0.03°, −8%, p = 0.28) and to 0.28° in flexion–extension (−0.12°, −30%, p = 0.51), (Fig. 4A).

The median value of pedicle screw rotation in the configuration without crosslink was 0.56° in axial rotation, 1.44° in lateral bending and 0.39° in flexion–extension. The addition of a crosslink reduced this values to 0.29° in axial rotation (−0.27°, −48%, p = 0.02) and to 1.25° in lateral bending (−0.19°, −13%, p = 0.68). No reduction was measured in flexion–extension (−0°, −0%, p = 0.77) (Fig. 4B).

Comparing the two trajectories, the measured values for parallelogram deformation and screw rotation did not differ in a statistical significant way, except for the comparison of parallelogram deformation during lateral bending loading, for which parallelogram deformation was smaller in CBT-instrumented segments (Table 2). The absolute effect of crosslink-augmentation did not differ significantly between the two trajectories for both parallelogram deformation and screw rotation (Table 2).

**Table 2:** Comparison of parallelogram deformation and screw rotation in segments instrumented with the traditional trajectory (TT) and segments instrumented with the cortical bone trajectory (CBT).

		Without crosslink			Effect of crosslink		
	Loading	TT	CBT	p-value	TT	CBT	p-value
Parallelogram deformation	AR	2.76° (0.97°, 2.84°)	2.39° (1.37°, 5.24°)	0.69	0.94° (0.39°, 1.40°)	0.88° (0.58°, 2.51°)	0.45
	LB	0.36° (0.10°, 0.38°)	0.63° (0.26°, 1.13°)	0.02	0.03° (−0.17°, 0.29°)	0.11° (−0.12°, 0.59°)	0.45
	FE	0.41° (0.11°, 1.01°)	0.33° (0.08°, 0.87°)	0.69	0.26° (−0.15°, 0.60°)	−0.03° (−0.34°, 0.33°)	0.52
Screw rotation	AR	0.75° (0.24°, 1.18°)	0.54° (0.21°, 1.63°)	0.69	0.48° (−0.15°, 0.73°)	0.23° (−0.23°, 1.40°)	0.86
	LB	1.25° (0.88°, 2.52°)	1.60° (0.66°, 1.89°)	0.60	0.08° (−0.23°, 0.31°)	0.10° (−0.63°, 0.55°)	0.95
	FE	0.40° (0.30°, 0.71°)	0.26° (0.15°, 0.54°)	0.06	−0.04° (−0.44°, 0.26°)	0.01° (−0.31°, 0.14°)	0.86

The median (minimum, maximum) values are reported for each group.

*AR* axial rotation, *LB* lateral bending, *FE* flexion–extension.

## Discussion

After posterior screw-rod instrumentations, notable segmental motion can persist. Deformation of bone and implants can potentially allow for some “screw rotation” in the bone and with the two sides of the construct not being connected, relative movement can occur resulting in a “parallelogram-deformation” of the construct. By connecting the two sides, crosslinks are expected to reduce this effect. The aim of this study was to quantify the effect of crosslinks on the posterior screw-rod constructs by measuring screw rotation and parallelogram deformation in human cadavers during axial rotation, lateral bending, and flexion–extension loading.

Without crosslink, some screw rotation and comparably large parallelogram deformation were recorded during axial rotation. Axial rotation generates lateral shear forces at the posteriorly located screw-rod-construct, which we hypothesize to be the leading cause for the observed parallelogram deformation. The addition of a crosslink reduces this parallelogram deformation by about 60% and can explain the effectiveness of crosslink-augmentation in axial rotation^3^. We evaluate the remaining 40% of parallelogram deformation to be the result of elastic deformation of the screw-rod construct and the vertebral bodies under the acting loads, as no signs for plastic deformation of the instrumentation and no signs of screw loosening in the bone were observed. We evaluate the recorded screw rotation to be the direct result of the parallelogram deformation in this loading direction. By reducing the parallelogram deformation, crosslink-augmentation is therefore indirectly able to reduce screw rotation under these loading conditions.

In contrast, large screw rotation and only minimal parallelogram deformation were observed during lateral bending loading. The large screw rotation can be an important factor for the relatively large motion of an instrumented segment in this loading situation (Fig. 1A^1^). In this loading condition, the addition of a crosslink is no effective measure to reduce screw rotation, as it is not primarily caused by parallelogram deformation. We postulate that the specific behavior of the construct is largely caused by the boundary conditions of the biomechanical test setup: in our case, the setup allows for coupled motion in the translational plane orthogonal to the loading axis, which prevents the build-up of large lateral shear forces and which results in pure bending moments around the loading axis. Without the generation of relevant lateral shear forces, only limited parallelogram deformation is generated, which explains the small parallelogram deformation in our results. However, in a setup that does not allow coupled motion in the translational plane orthogonal to the loading axis (constrained lateral bending), relevant lateral shear forces can occur, which can, in turn, result in larger parallelogram deformation. In such a situation, crosslink-augmentation could result in an increase in construct stiffness by reducing this parallelogram deformation. This consideration can help explain the large variability of the effect of crosslink-augmentation during lateral bending in the literature^3^. While we evaluate neither pure nor constrained bending moments to perfectly represent in-vivo loading conditions and while both boundary conditions can be useful for specific research questions, knowledge about the characteristics of the test setup is nevertheless crucial for the correct interpretation of biomechanical results.

In flexion–extension, only minimal screw rotation and very small parallelogram deformation were recorded, which can be explained by the small torque around the screw axes and the minimal lateral shear forces in this loading condition. In consequence, crosslink-augmentation is not able to affect construct stiffness in this loading condition, which is in line with the findings of the literature^3^.

The comparison of the two pedicle screw trajectories did not reveal significant differences in the effectiveness of crosslinks in regard to parallelogram deformation and screw rotation. This finding is in line with the similar effectiveness of crosslink-augmentation on segmental range of motion^1^ and does not lead to the conclusion that any of the two trajectories is more prone to benefit from crosslink augmentation in the clinical routine. Furthermore this finding allows for the combined analysis of all spinal segments, which helps achieve higher statistical power. While in the configuration without crosslink, parallelogram deformation was significantly larger in the CBT-instrumented segments, the absolute values were comparibly small for both trajectories. Therefore we evaluate the measured difference to be irrelevant for the clinical routine.

The here presented study is associated with certain limitations: screw rotation and parallelogram deformation were evaluated in the 2D-plane of the telecentric objective. While this plane was oriented in the coronal plane of the segments, small variability between loading conditions and between specimens cannot be excluded. Also, some inaccuracy during image processing must be assumed, which increases the variability of the results. Furthermore, the experiments were conducted on cadaveric specimens at room temperature loaded with well-defined but largely simplified loading conditions compared to the in-vivo situation. These aspects limit the direct transferability of the findings to the clinical situation. Nevertheless, we evaluate the qualitative conclusions of this study to be robust against these potential sources of error. Finally, a rather simple configuration with only single-level instrumentations without additional decompression procedures or interbody cage insertion was chosen to keep additional parameters minimal. This experimental setting limits definitve conclusions on more complex configurations.

## Conclusion

In axial rotation, crosslink-augmentation can reduce parallelogram deformation and with that, screw rotation. This explains the effectiveness of crosslinks in this loading condition. In unconstrained lateral bending and flexion–extension, parallelogram deformation is minimal and therefore, crosslink-augmentation has no significant effect. Since the relatively large screw rotation in lateral bending is not caused by parallelogram deformation, crosslink-augmentation is no adequate countermeasure for this problem. The fundamental understanding and the quantitative information about the biomechanical effect of crosslink-augmentation on construct stiffness and construct deformation helps to evaluate its potential benefit in the clinical practice and to better understand the limitations of its effectiveness.

## Supplementary Information

Supplementary Information.

Supplementary Legends.
