# Supplementary material for: The biomechanical fundamentals of crosslink-augmentation in posterior spinal instrumentation
Source: Sci Rep. 2022 May 10;12:7621. doi: 10.1038/s41598-022-11719-2 (PMC9090827; doi:10.1038/s41598-022-11719-2)
Supplement: Supplementary file 2 — Supplementary Legends. [file 41598_2022_11719_MOESM2_ESM.docx]

SUPPLEMENTARY LEGENDS

Title: Table of Results

Legend: The parallelogram deformation and screw rotation values for each specimen, for both configurations and for all three loading conditions are listed. FE_NoCL = Flexion-Extension loading without crosslink-augmentation, FE_CL = Flexion-Extension loading with crosslink-augmentation, LB_NoCL = Lateral bending loading without crosslink-augmentation, LB_CL = Lateral bending loading without crosslink-augmentation, AR_NoCL = Axial rotation loading without crosslink-augmentation, AR_CL = Axial rotation loading with crosslink-augmentation
